# Analysis of Novel Drug-Resistant Human Cytomegalovirus DNA Polymerase Mutations Reveals the Role of a DNA-Binding Loop in Phosphonoformic Acid Resistance

**DOI:** 10.3389/fmicb.2022.771978

**Published:** 2022-02-03

**Authors:** Kye Ryeong Park, Young-Eui Kim, Amen Shamim, Shuang Gong, Soo-Han Choi, Kyeong Kyu Kim, Yae-Jean Kim, Jin-Hyun Ahn

**Affiliations:** ^1^Department of Microbiology, Sungkyunkwan University School of Medicine, Suwon, South Korea; ^2^Department of Precision Medicine, Institute for Antimicrobial Resistance Research and Therapeutics, Sungkyunkwan University School of Medicine, Suwon, South Korea; ^3^Department of Computer Science, University of Agriculture, Faisalabad, Pakistan; ^4^Department of Pediatrics, Pusan National University Hospital, Busan, South Korea; ^5^Samsung Biomedical Research Institute, Samsung Medical Center, Seoul, South Korea; ^6^Department of Pediatrics, Samsung Medical Center, Sungkyunkwan University School of Medicine, Seoul, South Korea

**Keywords:** HCMV, polymerase, drug, phosphonoacetic acid, mutation

## Abstract

The appearance of drug-resistant mutations in UL54 DNA polymerase and UL97 kinase genes is problematic for the treatment of human cytomegalovirus (HCMV) diseases. During treatment of HCMV infection in a pediatric hematopoietic cell transplant recipient, H600L and T700A mutations and E576G mutation were independently found in the UL54 gene. Foscarnet (FOS; phosphonoformic acid) resistance by T700A mutation is reported. Here, we investigated the role of novel mutations in drug resistance by producing recombinant viruses and a model polymerase structure. The H600L mutant virus showed an increase in resistance to ganciclovir (GCV) by 11-fold and to FOS and cidofovir (CDV) by 5-fold, compared to the wild type, while the E756G mutant virus showed an increase in resistance to FOS by 9-fold and modestly to CDV by 2-fold. With the FOS-resistant T700A mutation, only H600L produced increased FOS resistance up to 37-fold, indicating an additive effect of these mutations on FOS resistance. To gain insight into drug resistance mechanisms, a model structure for UL54 polymerase was constructed using the yeast DNA polymerase as a template. In this model, HCMV DNA polymerase contains a long palm loop domain of which H600 and T700 are located on each end and T700 interacts with the FOS binding pocket. Our results demonstrate that H600L and E756G mutations in UL54 polymerase are novel drug-resistant mutations and that the acquisition of both H600L and T700A mutations in the DNA-binding loop confers increased resistance to FOS treatment, providing novel insights for the mechanism acquiring foscarnet resistance.

## Introduction

Human cytomegalovirus (HCMV) infection is a leading cause of morbidity in allogeneic hematopoietic cell transplant (alloHCT) recipients (Ljungman et al., 2002; Boeckh and Ljungman, 2009). Antiviral drugs targeting viral DNA polymerase, such as ganciclovir (GCV), foscarnet (FOS), and cidofovir (CDV), and the prodrug valganciclovir (VGCV) have been used for the treatment of HCMV disease (Andrei et al., 2009). GCV is a nucleoside analogue of guanosine, CDV is a non-cyclic analogue of cytidine monophosphate, and VGCV is an L-valyl ester prodrug of GCV. FOS (phosphonoformic acid; PFA), which is structurally similar to the pyrophosphate anion, binds to the HCMV UL54 DNA polymerase and blocks the pyrophosphate-binding site, preventing the incorporation of incoming deoxynucleotide triphosphates (dNTPs) into viral DNA (Chrisp and Clissold, 1991). Recently, letermovir, an inhibitor of the viral terminase complex, has been approved for use as a prophylactic agent (Marty et al., 2017; Foolad et al., 2018).

Prolonged exposure of HCMV to drugs often leads to the appearance of drug-resistant mutant viruses. For drugs acting on viral DNA polymerase, the mutations are found in the UL54 gene encoding HCMV DNA polymerase and the UL97 gene encoding viral kinase. Several drug-resistant UL54 mutations confer resistance to FOS or CDV, or multi-drugs, such as GCV/CDV, GCV/FOS, or GCV/CDV/FOS, while drug-resistant UL97 mutations are known to confer resistance to GCV only (for review, Chou, 2020). Multi-drug resistance may arise from a single HCMV containing multiple mutations or mixed infection of viruses containing different mutations (Chou et al., 2003). Drug-resistant mutations to letermovir and mutations to maribavir, an inhibitor of viral pUL97 kinase, have been reported (Piret and Boivin, 2019).

During treatment of a pediatric alloHSCT recipient, we identified UL54 mutations including novel H600L and E756G mutations (Choi et al., 2014). The H600L mutation occurs between the conserved δC region and region II in the central polymerization domain of the HCMV DNA polymerase, while E756G occurs between the regions II and III (Lurain and Chou, 2010). To address the role of these newly identified mutations for drug resistance, it is necessary to produce recombinant viruses containing the mutations and assess their drug phenotype (Lurain and Chou, 2010). In this study, we examined the role of H600L and E576G viral polymerase mutations in GCV, FOS, and CDV resistance by producing recombinant viruses and testing their drug phenotype. We also studied the drug resistance mechanism as a result of these mutations by producing a model structure of UL54 polymerase.

## Materials and Methods

### Cell Culture, Viruses, and Chemicals

Human foreskin fibroblast (HF) cells were grown in Dulbecco’s modified Eagle’s medium supplemented with 10% fetal bovine serum, penicillin (100 U/ml), and streptomycin (100 μg/ml) in a 5% CO_2_ humidified incubator at 37°C. The titers of HCMV were determined as infectious units (IFUs) after measurement of IE1-positive cells in infectious center assays or plaque forming units (PFUs) using HF cells. The HCMV (Toledo) viral stocks were prepared and used according to previously described methods (Kwon et al., 2017). GCV, FOS, and CDV were purchased from Roche, Fresenius Kabi, and Sigma-Aldrich, respectively.

### Site-Directed Mutagenesis of the UL54 Gene

The UL54 DNA fragment encompassing amino acids from 564 to 794 was PCR-amplified with primers (LMV1885/1886). The amplified PCR products were digested with *Eco*RI and *Pst*I and cloned into pUL18 (NEB). For site-directed mutagenesis, pUC18-UL54 was used as a PCR-based mutagenesis template. The mutagenesis primers were LMV1877/1878 (for H600L mutation), LMV1881/1882 (T700A), and LMV1883/1884 (E756G). The sequences of primers used for UL54 mutagenesis are listed in Table 1.

**Table 1:** Oligonucleotide primers used in this study.

LMV1808	5'-TCACCCACACTGTGCCCATCTACGA-3'
LMV1809	5'-CAGCGGAACCGCTCATTGCCAATGG-3'
LMV1885	5'-GATGAATTCCGGCTGGCTAAAATTCCGTTG-3'
LMV1886	5'-GATCTGCAGGTCTTGACACTCGCGCATGCA-3'
LMV1877	5'-CTCCCCAACCTCTACAGCAAA-3'
LMV1878	5'-TTTGCTGTAGAGGTTGGGCAG-3'
LMV1881	5'-CCAGGGCGCCGCGGTGTTTGA-3'
LMV1882	5'-TCAAACACCGCGGCGCCCTGG-3'
LMV1883	5'-GTCACGCTAGGGAACGGCGTG-3'
LMV1884	5'-CACGCCGTTCCCTAGCGTGAC-3'
LMV1890	5'-AGCAGATCCGTATCTACACCTCGCTGCTGGACGAGTGCGCCTGCCGCGATGGCCTGGTGATGATGGCGGGATCG-3'
LMV1891	5'-AGTTCCGAAAGCACCGAGACGCGCACCGAAGCACGCACAAAGCGGTGTGTTCAGAAGAACTCGTCAAGAAGGCG-3'
LMV1892	5'-AGCAGATCCGTATCTACACC-3'
LMV1893	5'-AGTTCCGAAAGCACCGAGAC-3'
LMV2343	5'-GCAAAAGGCGCAGTTTTCTA-3'
LMV2344	5'-TCCTACCCTGTCTCCACCAC-3'

### Bacmid Mutagenesis

The HCMV (Toledo) bacmid (Murphy et al., 2003) was provided by Hua Zhu (UMDNJ-New Jersey Medical School, Newark, NJ, United States) and used as a template to introduce UL54 mutations using a counter-selection bacterial artificial chromosome (BAC) modification kit (Gene Bridges). Briefly, *rpsL-neo* cassettes flanked by homology arms with 100 nucleotides of the region upstream and downstream of the target site were amplified using primers (LMV1890/1891). The amplified *rpsL-neo* fragments were purified and introduced into *Escherichia coli* DH10B containing the wild-type bacmid for recombination *via* electroporation using Gene Pulser II (Bio-Rad). The intermediate bacmid construct containing the *rpsL-neo* cassette was selected on Luria Bertani (LB) agar plates containing kanamycin. Next, the mutant UL54 fragments for replacing the *rpsL-neo* cassette were PCR-amplified with primers (LMV1892/1893) from pUC18-UL54 plasmids and recombined into the bacmid DNA containing the *rpsL-neo* cassette. *Escherichia coli* cells containing the mutant UL54 bacmid were selected on LB plates containing streptomycin. The mutation regions were amplified by PCR and sequenced to verify the desired mutations. The primers used for bacmid mutagenesis are listed in Table 1.

### Production of Recombinant Viruses From Bacmids

Bacmid DNA was introduced to HF cells *via* electroporation. For each reaction, HF cells (2 × 10^6^) in 300 μl R buffer were mixed with 5 μg Toledo-BAC DNA, 3 μg pCMV71 encoding pp71, and 1 μg plasmid pEGFP-C1 for monitoring efficiency. Following electroporation at 1,350 V for 30 ms using a MP-100 microporator (Invitrogen), the cells were transferred to T-25 flasks. When they reached confluence, the cells were split into new T-75 flask cultures. The titer of virus stocks was determined by infect center assays. The mutation regions were PCR-amplified from the DNA isolated from the virus stocks and sequenced to confirm the desired mutations.

### Infectious Center Assays

HF cells in 24-well plates (1 × 10^5^ cells per well) were inoculated with diluted virus samples. At 24 h after infection, cells were fixed with 500 μl of cold methanol for 10 min. Cells were then washed three times in PBS and incubated with anti-IE1 rabbit polyclonal antibody in PBS at 37°C for 1 h, followed by incubation with phosphatase-conjugated anti-rabbit immunoglobulin G (IgG) antibody in PBS at 37°C for 1 h. After gentle wash in PBS, cells were treated with 200 μl of developing solution (nitro blue tetrazolium/5-bromo-4-chloro-3-indolylphosphate) at room temperature for 1 h. Positively stained cells were counted in five separate fields per well under a light microscope (×200 magnification).

### Plaque Assays

HF cells in 24-well plates (1.5 × 10^5^ cells per well) were mock-infected or infected with recombinant viruses at an MOI of 0.01. Following 1 h adsorption, the inoculum was removed and washed with phosphate-buffered saline (PBS). Cells were overlaid with an agarose medium consisting of DMEM with 2% FBS, 0.25% agarose, 100 unit/ml penicillin, and 100 μg/ml streptomycin, or medium containing serial GCV, FOS, or CDV concentrations. After 9 days, cells were fixed overnight with 10% formalin and the overlay was removed. The cell monolayer was then stained with 0.03% methylene blue and the number of plaques was counted. The effective concentration of drugs that reduce cytopathic effects by 50% (EC_50_) was calculated using GraphPad Prism software (version 5).

### Quantitation of Viral DNA

Total DNA was isolated from infected cells using the QIAamp DNA Mini kit (Qiagen) and eluted in 100 μl sterile water. Five microliters of elute was used for qPCR to measure the amount of viral DNA using the Power SYBR Green PCR Master Mix and QuantStudio™ Real-Time PCR System (Applied Biosystems). The LMV2343/2344 primers and LMV1808/1809 were used to amplify the UL75 gene for viral DNA quantitation and β-actin amplification for normalizing the threshold cycle (C_t_) values, respectively. The primers used for viral DNA quantitation are listed in Table 1.

### Modeling HCMV Polymerase Structure

The protein sequence of HCMV (Toledo strain) DNA polymerase (UL54) was obtained from UniPorts (D3YRZ3; residues 1–1,242). The crystal structure of the DNA polymerase δ (PDB ID 3IAY) of *Saccharomyces cerevisiae* (strain ATCC 204508/S288c) bound to DNA (Swan et al., 2009) was used as a template for modeling. The amino acid sequence alignment of the HCMV UL54 polymerase to the yeast DNA polymerase δ was prepared using ESPRIPT (Gouet et al., 2003). Homology modeling of HCMV polymerase was performed using MODELLER v9.22 (Webb and Sali, 2014). The best model was selected based on the MODELLER scoring function and its geometries, and contacts between amino acids were refined using energy minimization in UCSF Chimera (Pettersen et al., 2004). The final model was visualized in CCP4MG version 2.7.3 (McNicholas et al., 2011).

## Results

### Production of Recombinant Viruses Containing UL54 Mutations

In our previous prospective study, pediatric alloHCT recipients with HCMV infection who started preemptive antiviral therapy were enrolled for sequence analyses for UL54 and UL97 genes during the follow-up treatment period. In a patient who showed the first HCMV infection on post-HCT day 19 and the last antiviral therapy 19.8 months before enrollment, we found that mutations encoding H600L, T700A, and E756G occurred in the UL54 gene and a mutation encoding M460V occurred in the UL97 gene (Choi et al., 2014). H600L and T700A UL54 mutant viruses emerged during treatment with FOS, while the E756G UL54 mutant and the M460V UL97 mutant were selected during treatment with CDV plus GCV. Drug resistance based on the T700A or E756D/K/Q mutations in UL54 and M460V in UL97 have been reported (for review, Gohring et al., 2015).

**Figure 1:**
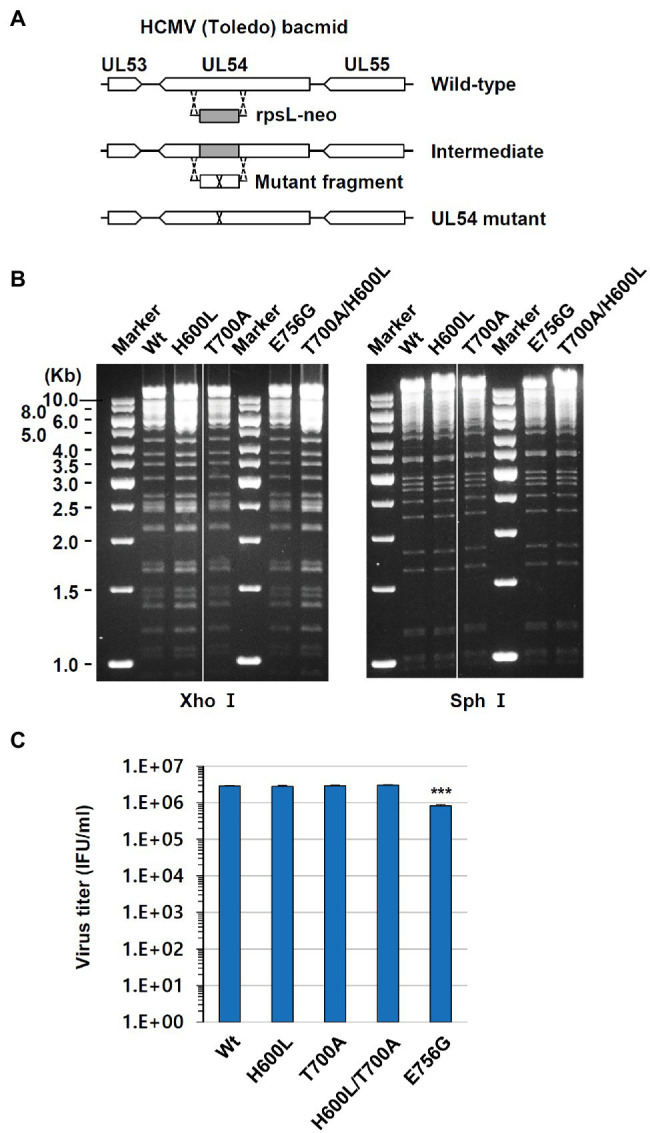
Production of human cytomegalovirus (HCMV) bacmids containing UL54 mutations. **(A)** Strategy for the production of HCMV bacmids containing UL54 mutations using a counter-selection BAC modification kit (see Materials and Methods). **(B)** Wild type and UL54 mutant bacmids were digested with *Xho*I or *Sph*I and subjected to agarose gel electrophoresis. **(C)** HF cells (1.5 × 10^5^ cell per well) in 12-well plates were infected with recombinant viruses containing the wild type or mutant UL54 gene at an MOI of 1 (IFU/ml) for 5 days. Progeny virus titers in the culture supernatants were measured by infectious center assays. Results shown are mean values and standard errors of triplicates. Statistical significance between the wide type and each mutant virus was determined using Student’s *t*-test. The values of *p* < .05 are considered statistically significant and the value of *p* < .001 (***) is indicated.

We investigated the role of the novel H600L and E756G mutations for drug resistance by producing recombinant viruses. We also included the T700A mutation, which was reported to confer FOS resistance (Baldanti et al., 1996; Cihlar et al., 1998), as a control in our study. HCMV (Toledo strain) bacmids containing the single UL54 mutation H600L, T700A, or E756G were produced using a counter-selection marker (see Materials and Methods; Figure 1A). The bacmid containing double mutations T700A/H600L was also produced. When bacmid DNA fragments obtained by *Xho*I or *Sph*I digestion were separated *via* agarose gel electrophoresis, the patterns of restriction enzyme-digested DNA fragments of the wild type and mutant bacmids did not show any apparent differences, indicating no gross alteration of the bacmid genomes (Figure 1B).

Recombinant viruses containing UL54 mutations were grown in HF cells that were transfected with bacmid DNAs *via* electroporation. To investigate whether the UL54 mutations introduced affect viral growth, HF cell was infected with viruses at an MOI of 1 for 120 h and progeny virus titers in the culture supernatants were determined by infectious center assays. We found that the titers of H600L, T700A, and H600L/T700A mutants were comparable to that of wild type, but the titer of E756G mutant was slightly reduced (Figure 1C).

### Drug Phenotyping of Novel UL54 Mutations With Recombinant Viruses

To investigate susceptibility of wild type and UL54 mutant viruses to anti-CMV drugs, HF cells were infected with serially diluted wild type or mutant viruses, overlaid with agarose medium with or without drugs, and assayed for plaque formation to determine EC_50_ values for GCV, FOS, and CDV drugs (Table 2). In our assays, EC_50_ values of GCV, FOS, and CDV in the wild-type virus in HF cells were 1.2, 34.3, and 0.1 μM, respectively, which are within the range of previously reported values (Chrisp and Clissold, 1991; McSharry et al., 2001). The T700A virus showed an 8-fold increased EC_50_ to FOS, but not to GCV and CDV, compared to the wild-type virus. This result is consistent with earlier reports demonstrating T700A as a FOS-resistant mutation (Baldanti et al., 1996; Cihlar et al., 1998). We found that the H600L mutant virus showed an increase in EC_50_ to GCV by 11-fold and to FOS and CDV by 5-fold, while the E756G mutant virus showed a significant increased EC_50_ to FOS by 9-fold and modestly to CDV by 2-fold. Notably, the H600L/T700A double-mutant virus showed an additional increase of FOS resistance up to 37-fold, whereas it did not show a significant increase of EC_50_ to GCV and CDV. These results of drug phenotyping with recombinant viruses identify H600L and E756G in UL54 as novel drug-resistant mutations and suggest that acquisition of both H600L and T700A mutations confers severe resistance to FOS treatment.

**Table 2:** Susceptibility of wild type and UL54 mutant viruses to GCV, FOS, and CDV drugs.

	Susceptibility					
	GCV		FOS		CDV	
Mutations	EC_50_^a^ (mean ± SD) (μM)	Fold increase^b^	EC_50_^a^ (mean ± SD) (μM)	Fold increase^b^	EC_50_^a^ (mean ± SD) (μM)	Fold increase^b^
Wild-type	1.18 ± .05	1.0	34.32 ± .22	1.0	.10 ± .09	1.0
H600L	13.14 ± .06	11.1	178.5 ± 3.34	5.2	.51 ± .08	4.9
T700A	1.06 ± .05	.9	261.2 ± .10	7.6	.07 ± .09	.7
E756G	1.14 ± .10	1.0	303.1 ± .33	8.8	.19 ± .08	1.8
H600L/T700A	1.66 ± .07	1.4	1,269 ± .23	37.0	.08 ± .06	.7

a EC_50_ is the effective concentration of antiviral drugs for 50% plaque reduction. Results are the mean ± SD of three determinations.

b Fold increase over that for the wild-type virus.

**Figure 2:**
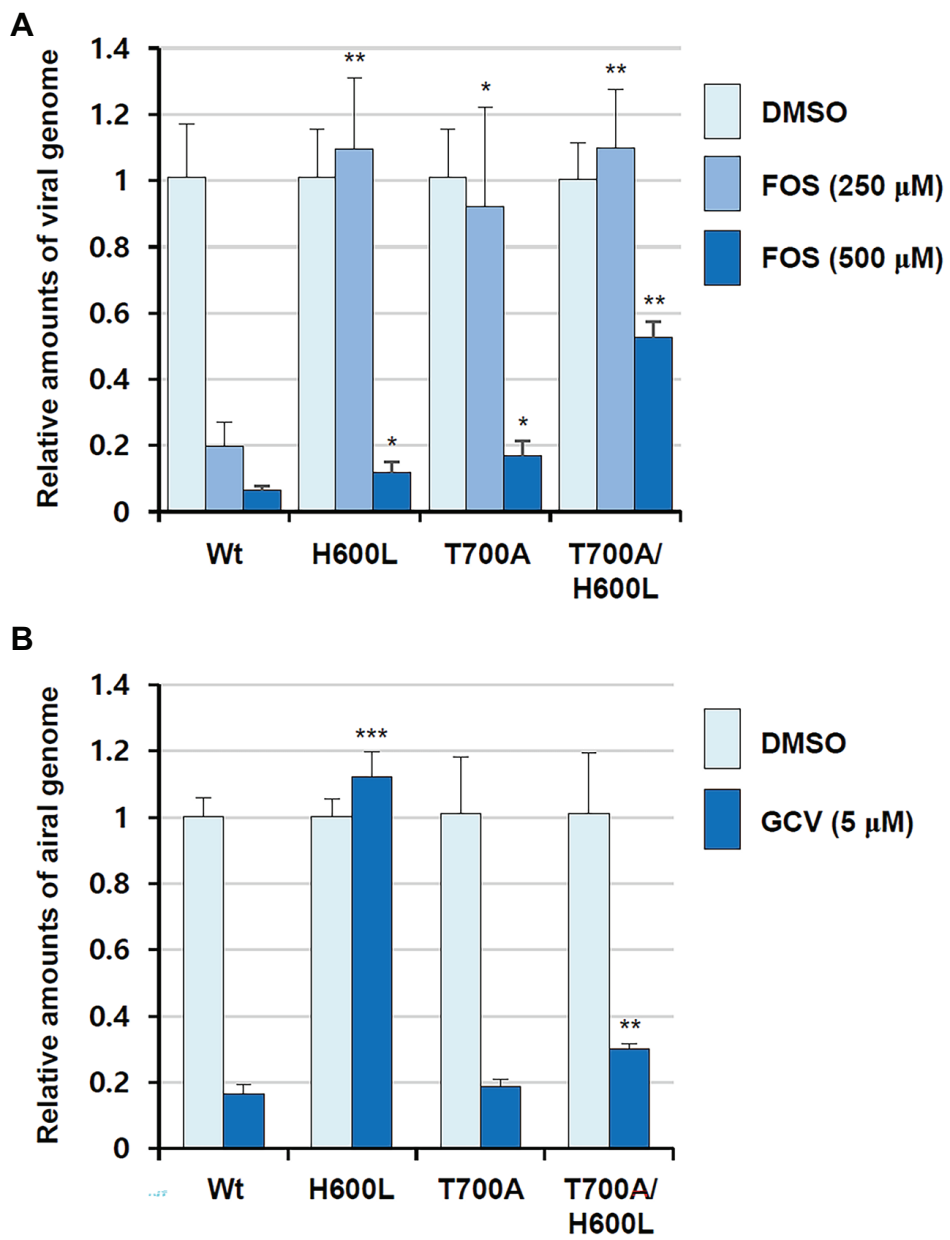
Effects of foscarnet (FOS) and ganciclovir (GCV) antivirals on viral DNA levels in wild type and UL54 mutant virus-infected cells. HF cells (2 × 10^5^) were infected with wild type or mutant (H600L, T700A, or H600L/T700A) viruses at an MOI of 0.1 (IFU/ml) as indicated and incubated for 6 days in the absence or presence of 250 or 500 μM FOS **(A)** or 5 μM GCV **(B)**. Total DNA was isolated in cells and the amount of viral DNA level was measured using qPCR (see Materials and Methods). Statistical significance was determined between the wide type and each mutant virus at given drug concentrations and the values of *p* < .05 (*), <.01 (**), or <.001 (***) are indicated.

To confirm the additive effect of H600L and T700A on FOS resistance, the effects of double H600L and T700A mutations on drug resistance were also confirmed by determining viral DNA levels. HF cells were infected with wide type or H600L, T700A, or H600L/T700A mutant viruses in the absence or presence of FOS, and the level of replicated viral DNA was measured using qPCR. When cells were treated with 250 μM FOS, all mutant viruses, but not the wild-type virus, replicated as effectively as in untreated cells. In cells treated with 500 μM FOS, the H600L/T700A double-mutant virus showed a higher viral DNA level than the wild type and other mutant viruses (Figure 2A). Although the DNA yield reduction EC_50_ for this double mutant appeared to be lower than the plaque reduction EC_50_, these results confirm that the H600L and T700A double mutant lead to an increased FOS resistance compared to single mutants. In a control experiment, the H600L virus effectively replicated in the presence of 5 μM GCV, compared to the wild type and other mutant viruses, as expected (Figure 2B).

### Positions of H600L, T700A, and E756G Mutations in a Polymerase Model Structure

**Figure 3:**
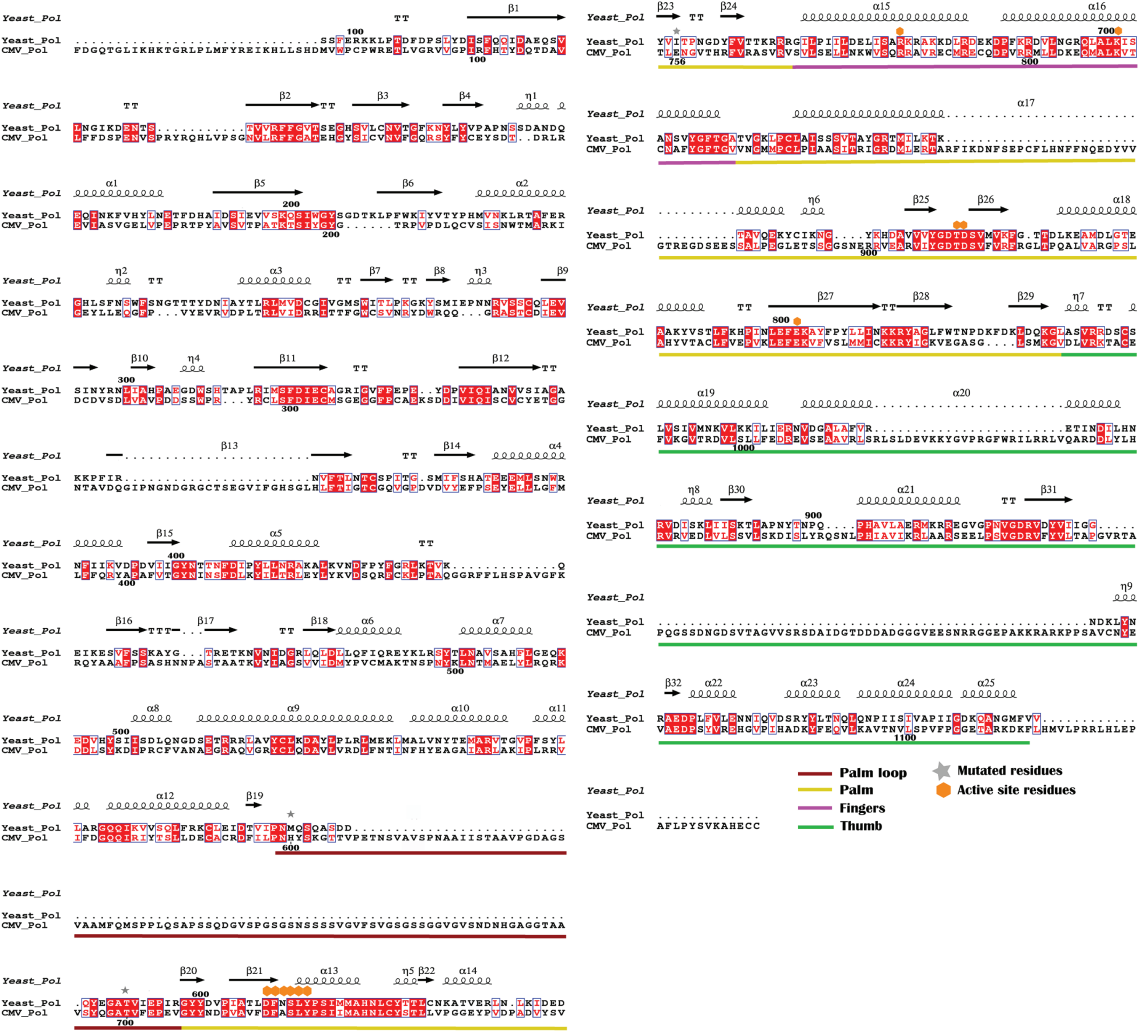
Sequence alignment of yeast DNA polymerase δ with HCMV DNA polymerase UL54. The secondary structure of yeast DNA polymerase is displayed along with the amino acid sequence. Identical residues are highlighted in red. Brick, yellow, pink, and green bars cover regions for the palm loop, palm, thumb, and finger domains, respectively. Mutant and active site resides are also indicated by star and diamond symbols, respectively.

To investigate the drug resistance mechanism of the mutations in this study, we modeled the three-dimensional structure of the HCMV UL54 DNA polymerase using the crystal structure of yeast DNA polymerase δ as a template for homology modeling since it has 32% sequence identity for 406 amino acids in the polymerase domain (696–1,101) of UL54 (Figure 3). The predicted UL54 model consists of three domains, palm (residues 600–770 and 824–979), finger (residues 771–823), and thumb (residues 980–1,166; Figure 3). The palm domain has mixed *β-sheets (β20–β29)* flanked by long *α-helices (α13, α14, α17*, and *α18)*. The finger domain consists of two curved antiparallel *α-helices* while the thumb domain is comprised of multiple *α-helices*.

**Figure 4:**
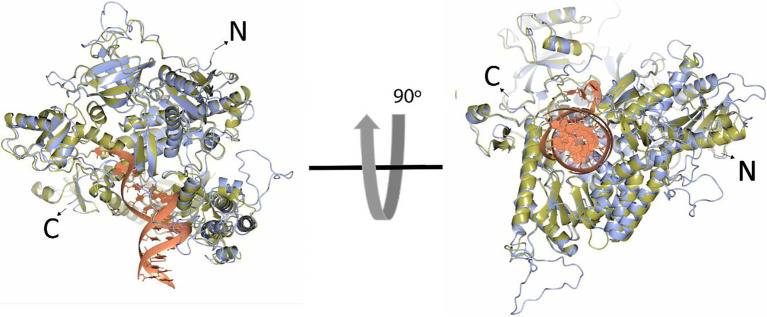
Superposition of the HCMV DNA polymerase and yeast DNA polymerase δ ribbon models. The ribbon models of HCMV DNA polymerase (blue) and the ternary complex of yeast DNA polymerase δ (yellow, Protein Data Bank ID 3iAY) are superimposed in two different orientations (90 degrees). The DNA double helix is shown in the orange ribbon model. N- and C-termini are indicated.

**Figure 5:**
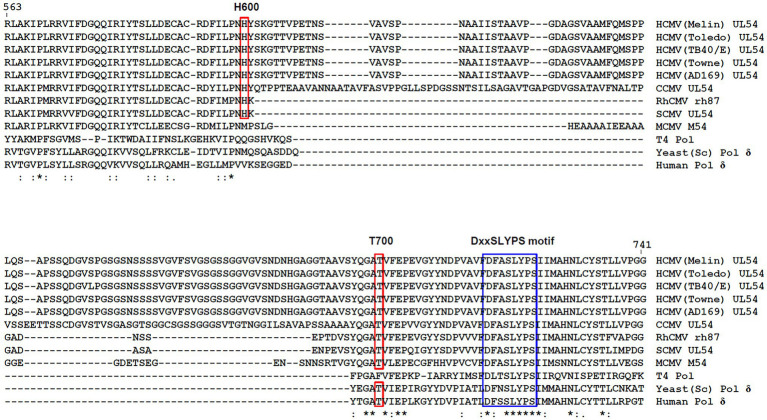
H600 and T700 amino acid positions in the palm loop of viral DNA polymerases. Amino acid sequences including H600 and T700 of HCMV (Toledo) UL54 are aligned with other sequences from other CMV polymerases, T4 polymerase, yeast polymerase δ, and human polymerase δ using CLUSTALW. Residue numbers in HCMV (Toledo) UL54 are shown. Identical (*), strongly similar (:), and weakly similar (.) sequences are indicated. The amino acid positions for H600 and T700 in HCMV (Toledo) UL54 and the corresponding conserved residues in other CMV polymerases and for the highly conserved DxxSLYPS motif are boxed. Accession numbers for CMV DNA polymerases are as follows: AAR316191, HCMV (Merlin) UL54; ADD391151, HCMV (Toledo) UL54; AGQ472841, HCMV (TB40/E) UL54; ACM480431, HCMV (Towne) UL54; ACL511341, HCMV (AD169) UL54; NP_6126981, CCMV UL54; YP_0681801, RhCMV rh87; YP_0049360301, SCMV UL54; CCE570611, MCMV M54; NP_0496621, T4 Pol; PJP092141, yeast (*Saccharomyces cerevisiae*) Pol δ; and AAA357681, human Pol δ. CCMV, chimpanzee CMV; RhCMV, rhesus monkey CMV; SCMV, simian CMV; and MCMV, murine CMV.

**Figure 6:**
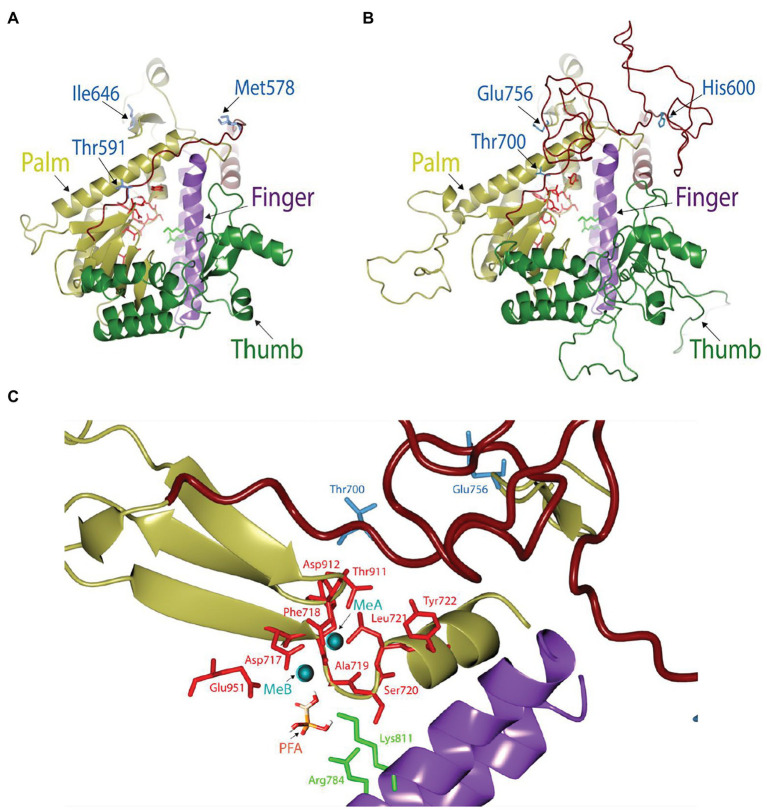
Structural view of the catalytic domain in HCMV DNA polymerase. **(A,B)** Comparison of the catalytic domains in yeast **(A)** and HCMV **(B)** DNA polymerases. Thumb (green), palm (yellow), and finger (purple) domains in each structure are drawn for comparison. The loop in the palm region is colored in brick. Key resides in the mutation study (blue), active site residues (red), and two basic residues involved in FOS binding in the finger domain (green) are displayed as stick models. **(C)** Close-up view of the FOS binding pocket of HCMV DNA polymerase in the presence of metal ions (MeA and MeB) and FOS. FOS (PFA) engages in electrostatic interaction with amino acid residues R784 and K811 (green) in the finger domain and also coordinate with metal ions (cyan) that are stabilized by acidic amino acid residues (red). Mutant site and active site amino acid residues are displayed as blue and red stick models, respectively.

In comparison to the yeast polymerase, the HCMV polymerase contains a long insertion (residues 608–694; Figures 3, 4), located near the initial part of palm, which is expected to form a long loop based on secondary structure prediction. Therefore, we named this region as a palm loop. Since this region cannot be modeled based on homology due to the lack of corresponding structure in the yeast polymerase, the MODELLER program loop modeling function was used to generate the model of this loop region. We found that this palm loop is present only in polymerases of HCMVs and chimpanzee CMV (CCMV), but not in polymerases of other primate CMVs or in the polymerase δ of yeast and human (Figure 5). When the drug-resistant mutation sites in this study were mapped in the structure, it was found that H600 is located near the N-terminal, and T700 is present in the C-terminal end of the palm loop region (Figures 5, 6A,B), suggesting that those residues might have a key role in controlling loop orientation or conformation. It is known that FOS binds to the pocket formed by basic residues in the finger domain and acidic residues in the palm domain (Zahn et al., 2011). Interestingly, T700 is located close to the active sites of UL54 and makes interaction with the FOS binding pocket (Figure 6C). Therefore, it is expected that the T700A mutant has lower binding affinity to FOS than the wild-type polymerase due to the conformational change caused by the mutation.

## Discussion

In the present study, we identified novel H600L and E756G mutations in the HCMV DNA polymerase UL54 as novel drug-resistant mutations. Drug phenotyping using recombinant viruses revealed that H600L confers FOS/GCV resistance, while E756G confers FOS/CDV resistance. FOS resistance evoked by change of E756 has been reported (Chou et al., 2000, 2008; Drouot et al., 2014). E756D and E756Q mutations led to FOS resistant, while E756K was resistant to FOS, GCV, and CDV. Of note, our data demonstrate that acquisition of H600L together with T700A, a previously known FOS-resistant mutation, confers severe resistance to FOS treatment, suggesting that H600L and T700A cooperate to evoke FOS resistance. In the patient, both H600L and T700A mutations seemed to appear after long exposure to FOS but disappeared when FOS was replaced with CDV, suggesting that these mutant viruses are sensitive to CDV (Choi et al., 2014). Consistent with that notion, the H600L/T700A double mutant did not show CDV resistance in our phenotyping analysis.

FOS, which is similar to the pyrophosphate anion, binds to the pyrophosphate-binding site of viral DNA polymerase and blocks incorporation of incoming dNTPs into viral DNA (Chrisp and Clissold, 1991). It has been suggested that the T700A mutation may affect DNA binding (Shi et al., 2006). The model structures of HCMV UL54 DNA polymerase have been previously reported based on the structures of bacteriophage RB69 DNA polymerases (Shi et al., 2006), herpes simplex virus-1 UL30 DNA polymerase apo enzyme, and yeast DNA polymerase δ complexed with DNA (Gilbert et al., 2011; Zarrouk et al., 2020). In our model structure separately generated based on the yeast polymerase structure (PDB ID 3IAY), we found that T700 is located in close proximity to the active sites of UL54 and interacts with the FOS binding pocket. Therefore, the conformational change induced by the T700A mutation is thought to reduce binding affinity to FOS. Interestingly, our model structure reveals that the HCMV polymerase has an additional long palm loop, of which H600 and T700 are located on each end. Therefore, we reason higher drug resistance of the double mutant at H600 and T700 is due to decreased FOS binding stability *via* affecting the conformational change of the palm loop, which may be involved in DNA accommodation as the part of the palm domain.

The results of this study suggest that alteration of DNA-binding regions is a common mode of acquiring FOS resistance. It is also notable that several family B polymerases, such as bacteriophage RB69 DNA polymerase (gp43) and the replicative eukaryotic DNA polymerases δ or ε, are not affected by FOS (Li et al., 2005) and that a chimeric RB69 DNA polymerase containing several substitutions of variable elements from UL54 acquires FOS sensitivity (Zahn et al., 2011). Among these substitutions, the V478W substitution, which corresponds to W780 in UL54, appears to be critical to push the finger domain to more readily adopt the closed conformation of the polymerase, making the chimeric DNA polymerase trapped in the untranslocated state and have increased affinity to FOS (Zahn et al., 2011). Therefore, it is also intriguing to speculate that modulation of DNA binding by H600L and T700A mutations in the palm domain affects the position of the finger domain.

## Data Availability Statement

The original contributions presented in the study are included in the article/supplementary material, further inquiries can be directed to the corresponding authors.

## Author Contributions

J-HA, Y-JK, and KK conceived and designed the study. KP, Y-EK, AS, and SG performed the experiments. J-HA, Y-JK, KK, and S-HC analyzed the data. J-HA and Y-JK wrote the manuscript. All authors contributed to the article and approved the submitted version.

## Funding

This work was supported by grants from the National Research Foundation of Korea (NRF) funded by the Ministry of Science and ICT (2019R1A2C2006676 and 2020R1A4A1018019 to J-HA and 2019R1A2C1009547 to Y-JK).

## Conflict of Interest

The authors declare that the research was conducted in the absence of any commercial or financial relationships that could be construed as a potential conflict of interest.

## Publisher’s Note

All claims expressed in this article are solely those of the authors and do not necessarily represent those of their affiliated organizations, or those of the publisher, the editors and the reviewers. Any product that may be evaluated in this article, or claim that may be made by its manufacturer, is not guaranteed or endorsed by the publisher.

## References

[ref1] Andrei G. De Clercq E. Snoeck R. (2009). Drug targets in cytomegalovirus infection. Infect. Disord. Drug Targets 9, 201–222. doi: 10.2174/187152609787847758 19275707

[ref2] Baldanti F. Underwood M. R. Stanat S. C. Biron K. K. Chou S. Sarasini A.. (1996). Single amino acid changes in the DNA polymerase confer foscarnet resistance and slow-growth phenotype, while mutations in the UL97-encoded phosphotransferase confer ganciclovir resistance in three double-resistant human cytomegalovirus strains recovered from patients with AIDS. J. Virol. 70, 1390–1395. doi: 10.1128/jvi.70.3.1390-1395.1996, PMID: 8627655 PMC189958

[ref3] Boeckh M. Ljungman P. (2009). How we treat cytomegalovirus in hematopoietic cell transplant recipients. Blood 113, 5711–5719. doi: 10.1182/blood-2008-10-143560, PMID: 19299333 PMC2700312

[ref4] Choi S. H. Hwang J. Y. Park K. S. Kim Y. Lee S. H. Yoo K. H.. (2014). The impact of drug-resistant cytomegalovirus in pediatric allogeneic hematopoietic cell transplant recipients: a prospective monitoring of UL97 and UL54 gene mutations. Transpl. Infect. Dis. 16, 919–929. doi: 10.1111/tid.12311, PMID: 25405808

[ref5] Chou S. (2020). Advances in the genotypic diagnosis of cytomegalovirus antiviral drug resistance. Antivir. Res. 176:104711. doi: 10.1016/j.antiviral.2020.104711, PMID: 31940472 PMC7080590

[ref6] Chou S. Lurain N. S. Thompson K. D. Miner R. C. Drew W. L. (2003). Viral DNA polymerase mutations associated with drug resistance in human cytomegalovirus. J. Infect. Dis. 188, 32–39. doi: 10.1086/375743 12825168

[ref7] Chou S. Marousek G. Li S. Weinberg A. (2008). Contrasting drug resistance phenotypes resulting from cytomegalovirus DNA polymerase mutations at the same exonuclease locus. J. Clin. Virol. 43, 107–109. doi: 10.1016/j.jcv.2008.04.005, PMID: 18502683 PMC2576411

[ref8] Chou S. Miner R. C. Drew W. L. (2000). A deletion mutation in region V of the cytomegalovirus DNA polymerase sequence confers multidrug resistance. J. Infect. Dis. 182, 1765–1768. doi: 10.1086/317618, PMID: 11069251

[ref9] Chrisp P. Clissold S. P. (1991). Foscarnet. A review of its antiviral activity, pharmacokinetic properties and therapeutic use in immunocompromised patients with cytomegalovirus retinitis. Drugs 41, 104–129. doi: 10.2165/00003495-199141010-00009 1706982

[ref10] Cihlar T. Fuller M. D. Cherrington J. M. (1998). Characterization of drug resistance-associated mutations in the human cytomegalovirus DNA polymerase gene by using recombinant mutant viruses generated from overlapping DNA fragments. J. Virol. 72, 5927–5936. doi: 10.1128/JVI.72.7.5927-5936.1998, PMID: 9621055 PMC110397

[ref11] Drouot E. Piret J. Lebel M. H. Boivin G. (2014). Characterization of multiple cytomegalovirus drug resistance mutations detected in a hematopoietic stem cell transplant recipient by recombinant phenotyping. J. Clin. Microbiol. 52, 4043–4046. doi: 10.1128/JCM.02205-14, PMID: 25143583 PMC4313248

[ref12] Foolad F. Aitken S. L. Chemaly R. F. (2018). Letermovir for the prevention of cytomegalovirus infection in adult cytomegalovirus-seropositive hematopoietic stem cell transplant recipients. Expert. Rev. Clin. Pharmacol. 11, 931–941. doi: 10.1080/17512433.2018.1500897, PMID: 30004790

[ref13] Gilbert C. Azzi A. Goyette N. Lin S. X. Boivin G. (2011). Recombinant phenotyping of cytomegalovirus UL54 mutations that emerged during cell passages in the presence of either ganciclovir or foscarnet. Antimicrob. Agents Chemother. 55, 4019–4027. doi: 10.1128/AAC.00334-11, PMID: 21709106 PMC3165324

[ref14] Gohring K. Hamprecht K. Jahn G. (2015). Antiviral drug- and multidrug resistance in cytomegalovirus infected SCT patients. Comput. Struct. Biotechnol. J. 13, 153–158. doi: 10.1016/j.csbj.2015.01.003, PMID: 25750703 PMC4348572

[ref15] Gouet P. Robert X. Courcelle E. (2003). ESPript/ENDscript: extracting and rendering sequence and 3D information from atomic structures of proteins. Nucleic Acids Res. 31, 3320–3323. doi: 10.1093/nar/gkg556, PMID: 12824317 PMC168963

[ref16] Kwon K. M. Oh S. E. Kim Y. E. Han T. H. Ahn J. H. (2017). Cooperative inhibition of RIP1-mediated NF-kappaB signaling by cytomegalovirus-encoded deubiquitinase and inactive homolog of cellular ribonucleotide reductase large subunit. PLoS Pathog. 13:e1006423. doi: 10.1371/journal.ppat.1006423, PMID: 28570668 PMC5469499

[ref17] Li L. Murphy K. M. Kanevets U. Reha-Krantz L. J. (2005). Sensitivity to phosphonoacetic acid: a new phenotype to probe DNA polymerase delta in *Saccharomyces cerevisiae*. Genetics 170, 569–580. doi: 10.1534/genetics.104.040295, PMID: 15802517 PMC1450396

[ref18] Ljungman P. Griffiths P. Paya C. (2002). Definitions of cytomegalovirus infection and disease in transplant recipients. Clin. Infect. Dis. 34, 1094–1097. doi: 10.1086/339329, PMID: 11914998

[ref19] Lurain N. S. Chou S. (2010). Antiviral drug resistance of human cytomegalovirus. Clin. Microbiol. Rev. 23, 689–712. doi: 10.1128/CMR.00009-10, PMID: 20930070 PMC2952978

[ref20] Marty F. M. Ljungman P. Chemaly R. F. Maertens J. Dadwal S. S. Duarte R. F.. (2017). Letermovir prophylaxis for cytomegalovirus in hematopoietic-cell transplantation. N. Engl. J. Med. 377, 2433–2444. doi: 10.1056/NEJMoa1706640 29211658

[ref21] McNicholas S. Potterton E. Wilson K. S. Noble M. E. (2011). Presenting your structures: the CCP4mg molecular-graphics software. Acta Crystallogr. D Biol. Crystallogr. 67, 386–394. doi: 10.1107/S0907444911007281, PMID: 21460457 PMC3069754

[ref22] McSharry J. J. Mcdonough A. Olson B. Hallenberger S. Reefschlaeger J. Bender W.. (2001). Susceptibilities of human cytomegalovirus clinical isolates to BAY38-4766, BAY43-9695, and ganciclovir. Antimicrob. Agents Chemother. 45, 2925–2927. doi: 10.1128/AAC.45.10.2925-2927.2001, PMID: 11557492 PMC90754

[ref23] Murphy E. Yu D. Grimwood J. Schmutz J. Dickson M. Jarvis M. A.. (2003). Coding potential of laboratory and clinical strains of human cytomegalovirus. Proc. Natl. Acad. Sci. U. S. A. 100, 14976–14981. doi: 10.1073/pnas.2136652100, PMID: 14657367 PMC299866

[ref24] Pettersen E. F. Goddard T. D. Huang C. C. Couch G. S. Greenblatt D. M. Meng E. C.. (2004). UCSF chimera: a visualization system for exploratory research and analysis. J. Comput. Chem. 25, 1605–1612. doi: 10.1002/jcc.20084, PMID: 15264254

[ref25] Piret J. Boivin G. (2019). Clinical development of letermovir and maribavir: overview of human cytomegalovirus drug resistance. Antivir. Res. 163, 91–105. doi: 10.1016/j.antiviral.2019.01.011, PMID: 30690043

[ref26] Shi R. Azzi A. Gilbert C. Boivin G. Lin S. X. (2006). Three-dimensional modeling of cytomegalovirus DNA polymerase and preliminary analysis of drug resistance. Proteins 64, 301–307. doi: 10.1002/prot.21005, PMID: 16705640

[ref27] Swan M. K. Johnson R. E. Prakash L. Prakash S. Aggarwal A. K. (2009). Structural basis of high-fidelity DNA synthesis by yeast DNA polymerase delta. Nat. Struct. Mol. Biol. 16, 979–986. doi: 10.1038/nsmb.1663, PMID: 19718023 PMC3055789

[ref28] Webb B. Sali A. (2014). Protein structure modeling with MODELLER. Methods Mol. Biol. 1137, 1–15. doi: 10.1007/978-1-4939-0366-5_1 24573470

[ref29] Zahn K. E. Tchesnokov E. P. Gotte M. Doublie S. (2011). Phosphonoformic acid inhibits viral replication by trapping the closed form of the DNA polymerase. J. Biol. Chem. 286, 25246–25255. doi: 10.1074/jbc.M111.248864, PMID: 21566148 PMC3137095

[ref30] Zarrouk K. Pham V. D. Piret J. Shi R. Boivin G. (2020). Hypersusceptibility of human cytomegalovirus to foscarnet induced by mutations in helices K and P of the viral DNA polymerase. Antimicrob. Agents Chemother. 64, e01910–e01919. doi: 10.1128/AAC.01910-19 32015044 PMC7179328

